# Baltic Sea management: Successes and failures

**DOI:** 10.1007/s13280-015-0653-9

**Published:** 2015-05-28

**Authors:** Ragnar Elmgren, Thorsten Blenckner, Agneta Andersson

**Affiliations:** Department of Ecology, Environment and Plant Sciences, Stockholm University, 106 91 Stockholm, Sweden; Stockholm Resilience Centre, Stockholm University, 106 91 Stockholm, Sweden; Department of Ecology and Environmental Science, Umeå University, 901 87 Umeå, Sweden

**Keywords:** Eutrophication, Persistent organic pollutants, Overfishing, Ecosystem-based management, Climate change

## Abstract

Severe environmental problems documented in the Baltic Sea in the 1960s led to the 1974 creation of the Helsinki Convention for the Protection of the Marine Environment of the Baltic Sea Area. We introduce this special issue by briefly summarizing successes and failures of Baltic environmental management in the following 40 years. The loads of many polluting substances have been greatly reduced, but legacy pollution slows recovery. Top predator populations have recovered, and human exposure to potential toxins has been reduced. The cod stock has partially recovered. Nutrient loads are decreasing, but deep-water anoxia and cyanobacterial blooms remain extensive, and climate change threatens the advances made. Ecosystem-based management is the agreed principle, but in practice the various environmental problems are still handled separately, since we still lack both basic ecological knowledge and appropriate governance structures for managing them together, in a true ecosystem approach.

## Introduction

**Fig. 1:**
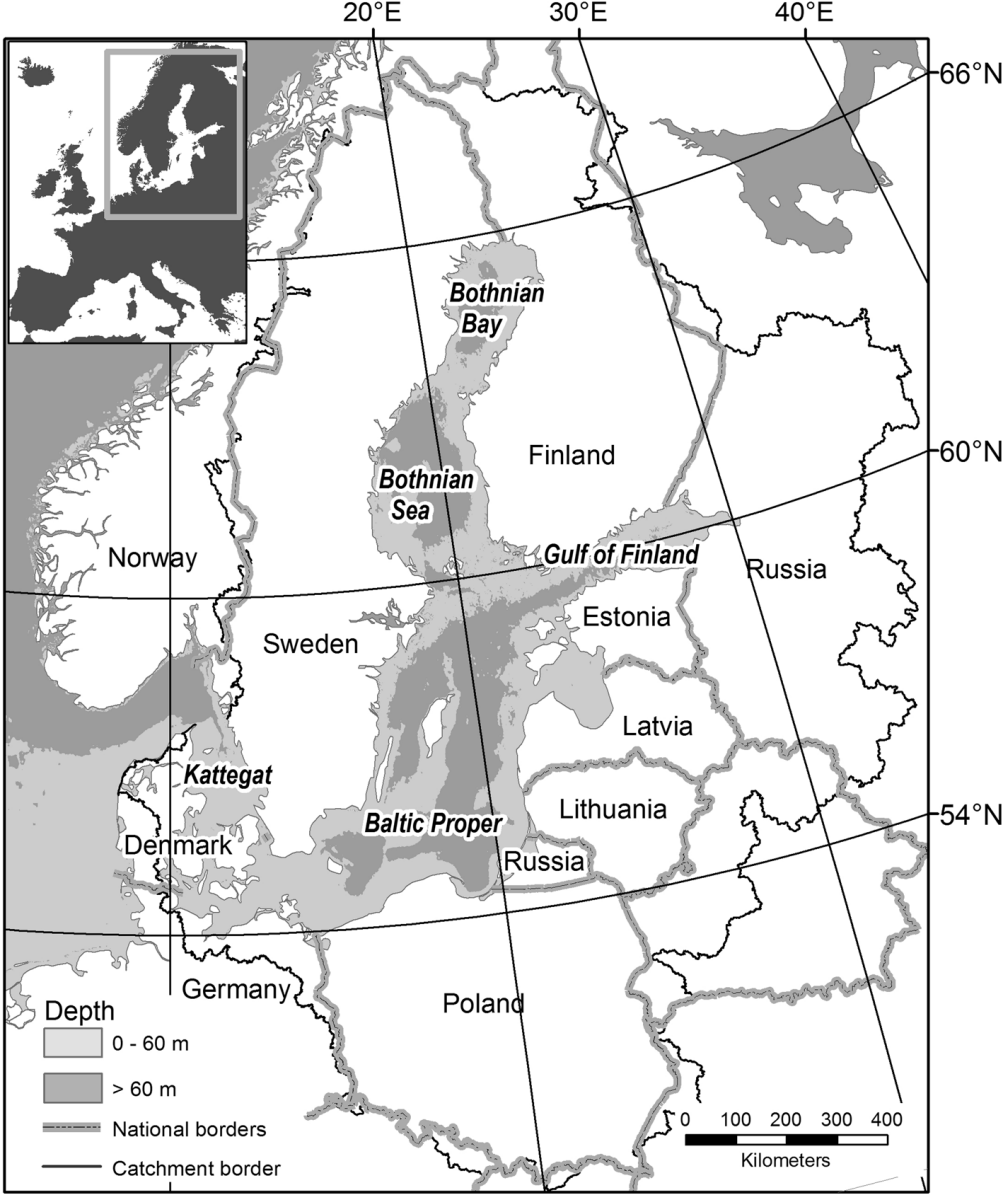
Baltic Sea map showing subareas, coastal nations, catchment border, and 60 m isobaths. Gulf of Bothnia = Bothnian Sea + Bothnian Bay

The Baltic Sea including the Kattegat (Fig. 1) is a large, almost non-tidal North European inland sea of 393 000 km^2^, with a mean depth of a mere 54 m. It is divided into a series of basins, mostly separated by shallow sounds or sills. Surface salinity gradually declines inwards, from 18–26 in the Kattegat to 2–4 in the innermost Bothnian Bay (using the unit-free Practical Salinity Scale, which is near-identical to older scales with units of g/kg or ‰). There is also permanent salinity stratification with depth, strongest in the Kattegat (bottom salinity 32–34) and weakening inwards to the Gulf of Bothnia. This causes stagnation of the bottom water, and in recent decades has led to widespread deep-water oxygen deficiency, seasonal in Kattegat and the Danish Sounds, near-permanent in the Baltic proper, intermittent in the Gulf of Finland, but not affecting the Gulf of Bothnia. Water renewal is slow, on the order of 50 years for the whole Baltic (description above based on Leppäranta and Myrberg 2009), making it vulnerable to pollution from the surrounding catchment (Fig. 1), with an area four times larger than the Baltic Sea, and a human population of some 85 million (Sweitzer et al. 1996). The waters of the Baltic Sea are generally cold, with the northern areas freezing over every winter, but the surface waters heat up in summer, in warm years to over 20 °C. These gradients cause large differences in ecological conditions along the sea, with biodiversity declining sharply with salinity (Remane and Schlieper 1971; Ojaveer et al. 2010).

**Fig. 2:**
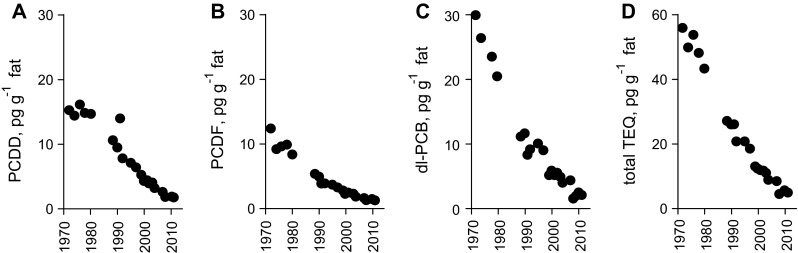
Decrease over time in mother’s milk from Sweden, 1972–2011, of **a** polychlorinated dibenzo-p-dioxins, **b** polychlorinated dibenzofurans, **c** dioxin-like polychlorinated biphenyls, **d** sum of toxic equivalents for PCDDs, PCDFs and dioxin-like PCBs (TEQ_WHO-2005_). Redrawn after Fång et al. (2013, Fig. 1)

The Baltic Sea is surrounded by nine riparian countries with a further five in the catchment, making its management politically complicated. Yet, it is perhaps the sea area with the longest record of scientific research and cooperation (Mills 1989), which from an early stage was expressly intended to support management of living marine resources. The International Council for the Exploration of the Sea (ICES), founded in 1901, was one of the first international intergovernmental scientific organizations and focused on the Baltic and North Seas and their living resources (Rozwadowski 2002). When environmental problems started to be seen as a threat to living marine resources in the late 1960s, an ICES working group documented severe environmental problems in the Baltic Sea (ICES 1970). These led to the formulation and 1974 signing of the Helsinki Convention for the Protection of the Marine Environment of the Baltic Sea Area, which is governed by the Helsinki Commission (HELCOM, http://www.helcom.fi/; Elmgren 2001). From the beginning, this convention had an emphasis on ecosystem management (Rozwadowski 2002), as was made formally explicit in 1992, when the convention was renegotiated after the fragmentation of the Soviet Union.

Since it was adopted by the Convention for Biological Diversity at the Rio Conference in 1992, ecosystem-based management (EBM) has become the norm for international conventions and organizations, such as ICES and the Food and Agriculture Organization of the United Nations (Leslie and McLeod 2007), as well as in EU’s Water and Marine Strategy Framework Directives (Anon. 2000, 2008). Accepting that EBM is the desired goal does not, however, without appropriate institutional basis and funding, guarantee its real life implementation (Österblom et al. 2010). In the early days, “the ecosystem approach” simply meant that the interactions of all components of the ecosystem, including humans, should be considered in the management process (e.g., Jansson 1972; Likens 1992), but starting with the Biodiversity Convention, EBM has come to signify a management process that also has social goals, such as an open and transparent democratic process with strong stakeholder participation, and an equitable distribution of the gains in human well-being resulting from improved management (Levin and Lubchenco 2008). The research on EBM has also increasingly become a search for indices and markers suitable for assessing progress for the political process (e.g., UNEP/GPA 2006; Curtin and Prezello 2010), rather than for a deeper understanding of ecosystem processes (cf. Thrush and Dayton 2010).

The four decades since 1974 seem a suitable period over which to evaluate successes and failures in the Helsinki Convention’s application of the ecosystem approach, aimed initially to “protect and enhance the environment of the Baltic Sea” (HELCOM 1993) and, after 1992, to “prevent and eliminate pollution in order to promote the ecological restoration of the Baltic Sea Area and the preservation of its ecological balance” (HELCOM 2008). In the following, we briefly describe some notable successes and failures in the management of the major environmental problems of the Baltic Sea, and assess how well the management process lives up to the ideals of EBM. We also discuss some new challenges for the future.

The two research programs this special issue reports on, Baltic Ecosystem Adaptive Management (BEAM) at Stockholm University and Ecosystem dynamics in the Baltic Sea in a changing climate perspective (ECOCHANGE) at Umeå and Linnaeus Universities, are examples of the type of interdisciplinary research needed to support EBM (Botey et al. 2014).

## Environmental successes and failures of the HELCOM period

### Toxic pollutants

The ICES (1970) report considered toxic pollutants as the foremost threat to the Baltic, with particular emphasis on heavy metals and DDT, but acknowledged that there was a large knowledge gap concerning other persistent organic pollutants (POPs). Based on toxicological experience from industrial exposure, heavy metals, also emitted to air and freshwater, were seen as posing a direct threat to humans. Industrial pollution, for example from pulp and paper mills, was commonplace and oil spills were frequent and often large, soiling shores and killing seabirds. Untreated sewage was an obvious problem in many enclosed coastal areas, especially near large cities, and some suspected that the recent increase in the area of oxygen-deficient bottom waters in the Baltic proper was also largely anthropogenic.

After 1974, the first decades of research described the basic functioning of the ecosystem (Jansson 1972; Elmgren 1984), and gave initial estimates of nutrient loads and effects (Larsson et al. 1985). International management was initially largely ineffective, due to political divisions, scientific uncertainty, and weak governance structures. The many problems of the Baltic Sea were tackled separately, often by entirely different management bodies; the fishery even by a separate convention, the Gdansk Convention (signed 1973). Still, use of a few problem pollutants, such as DDT (dichlorodiphenyltrichloroethane) and PCBs (polychlorinated biphenyls), was soon restricted or banned in the Baltic area. Concentrations of DDT, PCBs, dioxins, and dibenzofurans have since decreased rapidly in wildlife, demonstrating the effectiveness of mitigation (Nyberg et al. 2015). After a lag, previously highly threatened Baltic seal and white-tailed eagle (*Haliaeetus albicilla*) populations recovered rapidly, helped also by various conservation measures (Helander et al. 2008; Roos et al. 2012), visibly improving Baltic Sea biodiversity. Nevertheless, concentrations of dioxins and dioxin-like PCBs in fatty fish from parts of the Baltic remain higher than the maximum allowed in foodstuffs by EU. An exception from EU rules permits the sale of such fish for human consumption in Sweden and Finland, where they are the main route of human exposure to dioxin-like compounds. Even so, human exposure, as represented by the toxic equivalent levels of such compounds in mothers’ milk in Sweden, has decreased 6.5 % per year over a 40-year period from 1972 (Fig. 2; Fång et al. 2013), a truly remarkable improvement. Overall, the management of POPs is the greatest success story in Baltic environmental management but, for some groups of problem compounds, action has still been hesitant and tardy, e.g., for perfluorinated compounds (Roos et al. 2013) and many endocrine-disrupting chemicals (UNEP/WHO 2013).

Industrial pollution has also been curtailed, especially after the collapse of communism in Eastern Europe led to the closure of many heavily polluting and economically unviable plants, thus reducing heavy metal pollution (e.g., Vallius 2014). The HELCOM Joint Comprehensive Program of 1992 was specifically designed to identify and mitigate land-based “hot spots” of pollution (VanDeveer 2011). Still, legacy pollution maintains concentrations of some substances at levels of concern, e.g., PCBs (see above) and arsenic in the Bothnian Bay (Vallius 2014). Through introduction of double-hulled tankers, better surveillance and ship-routes, oil spills have been much reduced in frequency and volume (HELCOM Response 2013). Still, small, often intentional spills remain common and at an unfortunate time and place can kill thousands of seabirds. There have been few major spills from ship accidents in recent years, even though some consider that the risk of such spills may actually have increased due to large increases in ship traffic and the volume of oil transported on the Baltic Sea (Hassler 2010).

### Nutrient loads

The gradual accession of all coastal Baltic countries except Russia to the European Union by 2004 made important international environmental agreements legally binding in most of the HELCOM convention area and led to stronger action. Thus nutrient loads to the Baltic have been decreasing since c. 1980, with the largest reductions for phosphorus (Gustafsson et al. 2012), as countries have increasingly removed nutrients from municipal sewage, resulting in some notable local improvements in the coastal zone (e.g., Elmgren and Larsson 2001). Yet, in the Baltic proper, open-sea total nutrient concentrations have hardly decreased and anoxic bottom waters have affected record areas and volumes after 2000 (Carstensen et al. 2014). Winter concentrations of inorganic nitrogen have seen a slight decrease since the 1980s, but this may be due more to increased denitrification losses in the increased volume of anoxic bottom water, than to the load reduction (Vahtera et al. 2007). There are reports that primary production during the spring bloom has decreased somewhat, with a shift toward higher summer production (Raateoja et al. 2005, SW coast of Finland), accompanied by larger surface accumulations of cyanobacteria, as recorded by satellite (Kahru and Elmgren 2014, Baltic proper). The agreement on a Baltic Sea Action Plan in 2007 (revised 2013) was widely seen as a major step forward in Baltic Sea management, with nutrient reduction goals that were based on advanced ecosystem modeling (HELCOM 2007, 2013).

### Fishing

International Baltic Sea fishery management was initially weak, as exemplified by its inability to prevent overfishing of the Baltic cod (*Gadus morhua*) stock in the late 1980s, which led to large losses in income for the fishery (Sjöstrand 2008). The 2007 cod recovery plan (Anon. 2007), which for once has been adhered to by the politicians, has resulted in a considerable numerical recovery of the eastern cod stock. This success is only partial, however, since in combination with an increased minimum landing length and decreased availability of invertebrate prey through extensive oxygen deficiency, it has resulted in a large population of lean, slow-growing cod of reduced economic value that are harvested soon after reaching legal length (Svedäng and Hornborg 2014).

The Baltic Sea has a long history of mostly unintended introductions of non-indigenous species (Leppäkoski et al. 2002). HELCOM has followed this development, but, as in other marine areas, relatively little has yet been done to still the inflow of alien species. In the future, international agreements on the treatment of ballast water are likely to reduce, but hardly solve the problem, and species already established in the Baltic Sea are unlikely to be eliminated.

### Climate change

#### Invasive species

The Baltic Sea is one of the marine areas with the highest recorded temperature increases during the past century (Belkin 2009; Rutgersson et al. 2014), and this increase is almost certain to continue. A particularly important threshold will be reached once the winter temperature in the open Baltic proper remains above the temperature of maximum density. When this happens, above-halocline temperature convection may be much reduced, lowering both oxygen supply to the deep water and up-mixing of deep water, including pollutants and regenerated nutrients to the surface layer (Hordoir and Meier 2012). Since the causes of climate change are global, there is little HELCOM can do, except follow events and give advice on likely future developments and possibilities for adaptation. Global climate models, and the regional models based on them, generally agree that the Baltic will warm about 2–4 °C by the end of this century, but the projections vary more concerning effects on precipitation (Niiranen et al. 2013), which is likely to be particularly important in the Baltic Sea, where salinity is the ecological master factor (Elmgren and Hill 1997).

EBM must be adapted to the regional conditions, which vary widely in different basins of the Baltic Sea, as described above. Thus, eutrophication, anoxia, and overfishing are dominant problems in the Baltic proper, while arsenic and organic pollutants in fish and wildlife are of greater concern in the Gulf of Bothnia (e.g., Nyberg et al. 2015). On the whole, HELCOM has taken such regional differences into account, but it is worrying that the EU, in implementing the marine strategy directive, is trying to apply a single classification to the whole Baltic Sea area. HELCOM has also inspired and supported research on the Baltic Sea, without which the basis for decisions would have been much weaker, and the Helsinki Convention is now seen as the most successful of all international regional seas conventions.

### Holistic assessment

The Baltic Sea is often portrayed as an environmental disaster area, by the media, by non-governmental environmental organisations, and by some scientists. Even HELCOM (2010: Fig. 2.2, panel A), in its first holistic assessment of the ecosystem health of the Baltic Sea presented a map showing the whole Baltic Sea as having only moderate or (mostly) poor or bad ecosystem health, except for a small sliver in good health along the northern half of Sweden’s Gulf of Bothnia coast. This map has been produced from good scientific data, according to the principle of the water framework directive of “one out–all out,” and is in this sense correct. Yet it fails to convey the enormous value of the Baltic Sea still has as a natural resource for the millions living around it, even in its present somewhat tainted state. It also fails, crucially, to present the real progress that has been made, proving that investing in the environment pays off (Table 1). The remaining problems of the Baltic Sea are real and serious and will take a long time and large resources to mitigate (Table 1), but they would have been much worse without the environmental mitigation work coordinated by HELCOM and ICES. If the public is given the erroneous impression that 40 years of work and investment to protect the environment of the Baltic Sea has been a total failure, its support may falter (Elmgren et al. 2012).

**Table 1:** Evaluation of Baltic Sea Environmental Management in the HELCOM period

*Successes*	* Failures/remaining problems*
Helsinki Convention signed and ratified	HELCOM not organized for EBM
DDT, PCBs, TBT and other hazardous organics substances banned	Insufficient action on other endocrine-disrupting and perfluorinated compounds
Concentrations of dioxin-like compounds in wildlife greatly decreased Recovery of seal and eagle populations	Advice on human consumption and exemptions from standards still needed
Industrial heavy metal pollution reduced	Some “hot-spots” and legacy pollution remain
Oil spills reduced in frequency and volume	Seabirds still killed; major spill remains a risk
Nutrient loads considerably reduced, Baltic Sea Action Plan Coastal eutrophication status locally improved	Phosphorus concentration and anoxic volume at all-time high, cyanobacterial blooms larger
Cod recovery plan partial success	Cod concentrated in SW Baltic, grow slowly Fish catches not tailored to ecosystem needs Little effective action to prevent introduction of new invasive species Climate change continues apace
Ecosystem-based management approach enthusiastically adopted	Interconnected problems still managed apart; fisheries handled by a different convention
Water status classification system developed	Classification not adapted to climate change

## The case for ecosystem-based management

### A food-web perspective

EBM has become the accepted, if not yet the practiced, norm (Österblom et al. 2010; Möllmann et al. 2014). Yet, there are authors who maintain, with some good arguments, that EBM requires too much data and is too expensive to be used as a basis for fisheries management except perhaps for the largest stocks in the wealthiest countries (Hilborn and Ovando 2014), and may not always be needed even there (Cardinale and Svedäng 2011). Are the reasons for advocating EBM for the Baltic Sea really compelling? This requires that we can show that the different major problems of the Baltic Sea are so interconnected, that we cannot expect to deal effectively with them separately. No one disputes that fish in the sea ultimately depend on primary producers, in the open sea the phytoplankton, for their sustenance. Normally there is at least one trophic level between them, zooplankton that are eaten by small fish and fish larvae, but in many cases there are further intermediate trophic links, such as heterotrophic flagellates, ciliates, mysid shrimp, or benthic organisms, which all depend on the same primary production. It is therefore not surprising that catches of Baltic cod reached a maximum in the early 1980s, when Baltic eutrophication had become well established, but before the resulting expansion of oxygen deficiency had stopped recruitment from the spawning areas in Gdansk Bay and the eastern Gotland deep. Total yearly fisheries removals, on the other hand, peaked later, at about 1.5 million tons in 1998, as the depressed state of the overfished cod stock left more of their main fish prey, sprat (*Sprattus sprattus*) and herring (*Clupea harengus*), to the fishery (Zeller et al. 2011). Cod is far more valuable than the other major species in the Baltic fishery, herring and sprat, so the total value of the fishery increased initially as incipient eutrophication increased total fish production, only to fall again when oxygen deficiency became serious enough to stop the eastern spawning areas of cod from producing recruits.

Not only the quantity, but also the timing and taxonomy of the phytoplankton matter for fish and fisheries. The classic food chain for effectively supporting fish production is diatoms eaten by crustacean zooplankton eaten by fish. But in the Baltic Sea the main diatom bloom is the nitrogen-limited spring bloom, which is too early for extensive use by most crustacean zooplankton, and ends up largely sinking to the sea-floor (Elmgren 1978). This is followed by a summer-bloom of phosphorus-limited diazotrophic cyanobacteria, which has long been considered as low quality food for zooplankton. Recent research shows, however, that diazotrophic cyanobacteria release much of the nitrogen they fix to the water (Larsson et al. 2001; Ploug et al. 2010), effectively fertilizing it for other phytoplankton that bloom alongside them, such as small diatoms and picocyanobacteria. Eaten as part of a diet mixed with other phytoplankton, and the protozoans that feed on them, the diazotrophs can thus support rapid growth of crustacean zooplankton, at exactly the time of maximum food consumption by clupeid larvae and young-of-the-year (Karlson et al. 2015). This means that the summer cyanobacterial bloom is not only a nuisance for the tourist industry, but also supports fish recruitment in the Baltic, and that reducing the bloom may well carry a cost in lowered fish production.

The large freshwater inflow to the Gulf of Bothnia has high concentrations of colored organic matter, making the seawater brownish (Andersson et al. 2015). The organic input stimulates the heterotrophic microbial food web, but lowers pelagic and benthic primary production through shading and competition for production-limiting phosphorus (Ask et al. 2009; Wikner and Andersson 2012). The heterotrophic bacteria-based food web has one to two extra trophic levels (Berglund et al. 2007), making it less efficient than the phytoplankton-based food web. If climate change increases freshwater inflow to the Gulf of Bothnia as projected (Andersson et al. 2015), the pelagic food web may become even more bacteria-based, harming fish production and recruitment. On the other hand, a longer productive season and increased nutrient run-off may compensate, making the net effect of climate change difficult to predict (Andersson et al. 2015).

In recent years, it has become clear that fisheries management is central to managing the Baltic Sea environment, and that overfishing not only is bad business, and a threat to the fishing industry, but also a very serious environmental problem. Current fisheries management in the Baltic Sea is based on applying the principle of Maximum Sustainable Yield (MSY) to each fish stock separately. This principle has been applied globally for many fish stocks, often with positive results (Worm et al. 2009). Nevertheless, it is questionable on numerous counts. It is economically suboptimal in most cases, since the last fish is generally the most expensive to catch. This means that the maximum economic yield will normally be obtained by catching fewer fish than calculated from MSY. Fish catches need to vary less from year to year if the stock is larger, with more year-classes represented, again implying that catching less than under MSY is better economy. Further, MSY does not take changes in growth, in particular density-dependent growth, into account, with recent dire effects on the Eastern Baltic cod stock (Svedäng and Hornborg 2014). When several stocks with trophic interactions are fished, as in the Baltic Sea, MSY cannot simultaneously be attained for all stocks (e.g., Heath 2012). A multi-species maximum sustainable economic yield model might solve some of these problems, but would still not take the effects of fishing on the entire ecosystem fully into account (cf. Möllmann et al. 2014). Currently, the potential risk of trophic cascades from fish to primary producers is not taken into account when setting catch quotas. Such cascades are difficult to prove conclusively, but in the Baltic Sea proper they have been proposed as contributory causes both of increased phytoplankton blooms (Casini et al. 2008, 2012), and the decline of perennial coastal vegetation through over-growth by filamentous annual algae (Eriksson et al. 2011). In addition, overfishing of cod, by allowing stocks of sprat to increase, has probably been a major cause of a decline in growth rate of Baltic herring (Casini et al. 2010), such that herring in most of the Baltic Sea now take about 5–7 years to reach 40 g, whereas this took only 2–3 years around 1980 (ICES 2014). The older the fish, the more time it has had to accumulate organic contaminants (e.g., Kiljunen et al. 2007), indicating that cod overfishing has been a factor increasing human exposure to bioaccumulating POPs from the Baltic Sea, and in the continued ban, mentioned above, on sales of some Baltic fish outside Sweden and Finland. Eutrophication also influences the fate of organic pollutants in the Baltic Sea, with concentrations in fish expected to decrease when eutrophication increases, and vice versa (Larsson et al. 2000).

### Classification of water quality

For the implementation of the marine strategy framework directive, EU has developed a water status classification system focused on eutrophication and contaminants. This classification is largely based on estimated historical conditions, but considering ongoing and projected climate change, this may be inappropriate (McQuatters-Gollop 2012). For example, chlorophyll *a* in phytoplankton, which is an established quality factor in the classification, varies greatly with the light climate in the water. Through an increased export of colored dissolved organic matter (“brownification”), climate change is expected to lead to poorer light conditions in the Gulf of Bothnia, which will induce higher chlorophyll content in the phytoplankton community (Andersson and Rudehäll 1993), even without an increase in phytoplankton biomass. It is important that the classification system can adapt to such effects of climate alterations, or the water status may be incorrectly or inappropriately assessed.

### Governance

As we have illustrated above, the various environmental problems of the Baltic Sea are indeed so closely intertwined that we can hardly expect to deal successfully with them in a piecemeal fashion. Fish stocks depend on eutrophication, contaminants levels in seafood depend on fisheries management as well as on eutrophication, some negative effects of eutrophication can probably be mitigated by prudent fisheries management, and climate change will affect them all. The synergistic effects of the multiple drivers discussed above may cause unexpected changes in ecosystem functions, often called regime shifts, as reported from the Baltic Sea by Österblom et al. (2007). The potential for such abrupt changes in ecosystem state make it important to address several management sectors simultaneously, as in EBM (Levin and Möllmann 2015). The spatial aspect is of particular importance in geographically large and varied systems like the Baltic Sea, for example when cod management in one basin affects ecosystem dynamics in another basin (Casini et al. 2012).

Overall, we still lack both much of the basic ecological knowledge (Österblom et al. 2010) and the appropriate governance structures (Valman 2013) for effective implementation of EBM of the Baltic Sea. HELCOM was originally organized sector-by sector and the Baltic Sea Action plan was in part intended to re-organize the work to suit an ecosystem approach. However, Valman (2013) failed to find significant institutional change in HELCOM as a consequence of the Baltic Sea Action Plan, or, in fact, in the last 30 years. EBM requires not only ecological data collection, analysis, and modeling capacity, but also coordination between agencies and institutions across sectors and geographical levels. In addition to the top-down governance of the Baltic Sea by nation states through HELCOM and the European Council, there are also emerging bottom-up governance initiatives (Österblom et al. 2010). Experiments with local co-management illustrate the challenges and opportunities of collaborative, more inclusive forms of governance (Sandström et al. 2014). In all, we are still far from making management democratic and equitable, in an area of 14 independent countries, where people have widely diverging attitudes and aspirations and live under quite different economic and social conditions.

## Outlook

Prominent among the additional future challenges for Baltic Sea research and EBM is the controversial question of how large populations of top predators, such as seals and great cormorants (*Phalacrocorax carbo sinensis*), that should be maintained in the Baltic Sea, considering the possible cost in lowered fisheries yield, especially if eutrophication mitigation is successful, and results in lower production of commercial fish (cf. Zabel et al. 2003 for seals; KSLA 2013 for seals and cormorants). Climate change and perhaps marine acidification are also likely to change food web function, the interactions among environmental problems and, in the end, fish production (Meier et al. 2012; Lefébure et al. 2013; Niiranen et al. 2013). Environmental management decisions are increasingly being based on scenarios and simulation models, but these are simplifications of nature and no better than the imperfect knowledge on which they are based. Increasingly, managers want to base decisions on results of several independent models (ensemble modeling; Gårdmark et al. 2013; Meier et al. 2013). Models also often fail to incorporate mechanisms leading to sudden, unexpected regime shifts in the managed ecosystems (but see Niiranen et al. 2013). To minimize surprises, we need local and regional experimentation with management methods, coupled with adaptive management, where environmental monitoring is used to warn about sudden changes, or at least allows us to learn from them, so as not to be surprised the next time (Österblom et al. 2010).

For EBM to become successful, it must recognize that we are dealing with complex social-ecological systems that need governance mechanisms adapted to the scale of the problems, whether local, regional or global, with cooperative, multilevel management, partnership approaches, social learning, and knowledge co-production (Berkes 2012). EBM of the Baltic Sea will increasingly have to be extended to include also major human activities in the Baltic drainage basin, such as land use, and their incentive structures. To be realistic, we must also realize that implementing EBM is a ‘wicked problem,’ without simple, once for all solutions (Jentoft and Chuenpagdee 2009) and will require continuous adjustments, based on continued comprehensive environmental monitoring and gradually improved understanding through adaptive management and modeling (Levin and Möllmann 2015).

## Conclusions

Since the signing of the Helsinki convention 40 years ago, the Baltic Sea’s load of toxic pollutants has been greatly reduced; further eutrophication has been halted by greatly reducing nutrient emissions, and the numerical abundance of the main Baltic cod stock has recovered. A loss of the immense value of the Baltic Sea as a natural resource for the citizens of its coastal countries has been prevented. Nevertheless, many serious problems remain: potentially toxic pollutants are still at levels of concern both in wildlife and fish catches, and new ones continue to come into use; undesirable symptoms of eutrophication remain evident in many coastal areas; deep-water oxygen deficiency has record extension, and toxic blooms of cyanobacteria interfere too frequently with tourism and recreation. Little effective action has yet been taken to reduce the influx of non-indigenous, potentially invasive species, and fisheries management, while improved, still falls short. Finally, climate change proceeds at rapid pace, endangering the environmental gains that have been made, and making agreed goals harder to attain. EBM may already be the agreed principle, but it also needs to become the established practice!
